# Reactive cutaneous capillary endothelial proliferation in advanced hepatocellular carcinoma patients treated with camrelizumab: data derived from a multicenter phase 2 trial

**DOI:** 10.1186/s13045-020-00886-2

**Published:** 2020-05-11

**Authors:** Feng Wang, Shukui Qin, Xinchen Sun, Zhenggang Ren, Zhiqiang Meng, Zhendong Chen, Xiaoli Chai, Jianping Xiong, Yuxian Bai, Lin Yang, Hong Zhu, Weijia Fang, Xiaoyan Lin, Xiaoming Chen, Enxiao Li, Linna Wang, Ping Yan, Jianjun Zou

**Affiliations:** 1 Department of Radiotherapy, The First Affiliated Hospital of Nanjing Medical College, No. 300, Guangzhou Rode, Nanjing, 210029 China; 2 Department of Medical Oncology, Cancer Center of Jinling Hospital, No. 34, 34 Biao, Yanggongjing Street, Nanjing, 210002 China; 3 Liver Cancer Institute, Zhongshan Hospital, Fudan University, Shanghai, China; 4 Minimally Invasive Therapy Center, Fudan University Shanghai Cancer Center, Shanghai, China; 5 Department of Medical Oncology, The Second Affiliated Hospital of Anhui Medical University, Hefei, China; 6 Department of Intervention, Hunan Cancer Hospital, Changsha, China; 7 Department of Medical Oncology, The First Affiliated Hospital of Nanchang University, Nanchang, China; 8 Department of Medical Oncology, 3rd Affiliated Hospital of Harbin Medical University, Harbin, China; 9 Department of Medical Oncology, National Cancer Center/Cancer Hospital, Chinese Academy of Medical Sciences and Peking Union Medical College, Beijing, China; 10 Department of Medical Oncology, West China Hospital, Sichuan University, Chengdu, Sichuan China; 11 Department of Medical Oncology, The First Affiliated Hospital of Zhejiang University School of Medicine, Hangzhou, China; 12 Department of Medical Oncology, Fujian Medical University Union Hospital, Fuzhou, China; 13 Department of Interventional Radiology, Cancer Center, Guangdong Provincial People’s Hospital, Guangzhou, China; 14 Department of Medical Oncology, First Affiliated Hospital of Xi’an Jiaotong University (School of Medicine), Xi’an, China; 15 Clinical Research & Development, Jiangsu Hengrui Medicine Co., Ltd, Shanghai, China

**Keywords:** Anti–PD-1 antibody, Camrelizumab, Reactive cutaneous capillary endothelial proliferation, Hepatocellular carcinoma, Immune-related adverse events

## Abstract

**Background:**

Association of immune-related adverse events with tumor response has been reported. Reactive cutaneous capillary endothelial proliferation (RCCEP) is the most common adverse event related to camrelizumab, an immune checkpoint inhibitor, but lack of comprehensive analyses. In this study, we conducted comprehensive analyses on RCCEP in advanced hepatocellular carcinoma (HCC) patients treated with camrelizumab monotherapy.

**Methods:**

Data were derived from a Chinese nationwide, multicenter phase 2 trial of camrelizumab in pre-treated advanced HCC. The occurrence, clinicopathological characteristics, and prognostic value of RCCEP were analyzed.

**Results:**

With a median follow-up of 12.5 months, 145 of the 217 camrelizumab-treated patients (66.8%) experienced RCCEP (all grade 1 or 2). RCCEP occurred on the skin surface, mainly on the skin surface of head, face, and trunk. RCCEP could be divided into 5 types including “red-nevus-like,” “pearl-like,” “mulberry-like,” “patch-like,” and “tumor-like,” according to the morphological features. RCCEP biopsy and pathology showed capillary endothelial hyperplasia and capillary hyperplasia in dermis. Significant association between RCCEP occurrence with higher objective response rate was observed (19.3% vs. 5.6%; one-sided *p* = 0.0044). Compared with those without RCCEP, patients with RCCEP had prolonged progression-free survival (median PFS; 3.2 months vs. 1.9 months; one-sided *p* < 0.0001) and overall survival (median OS; 17.0 months vs. 5.8 months; one-sided *p* < 0.0001). In multivariable analyses, the development of RCCEP was significantly associated with prolonged PFS and OS after adjusting for baseline covariates. In addition, the landmark analyses of PFS and OS were consistent with the unadjusted analysis.

**Conclusions:**

RCCEP occurred on the skin surface and was an immune response of skin capillary endothelial cells. RCCEP occurrence positively associated with outcomes of camrelizumab in advanced HCC.

## Background

Immune checkpoint inhibitors (ICIs), especially monoclonal antibodies targeting programmed cell death protein-1 (PD-1) or its ligand PD-L1, have achieved remarkable results in the treatment of a variety of malignancies [1–5]. However, ICIs may cause immune-related adverse events (irAEs) due to overactivation of the body’s immune system, which has attracted great attention. IrAEs commonly occur in the skin, colon, endocrine organs, liver, and lung [6] and are mostly grade 1 or 2, but a few serious irAEs can lead to death [7]. Accordingly, several toxicity management guidelines for irAEs have been issued since 2017 [8–11].

Nivolumab and pembrolizumab achieved high and durable response with manageable safety profiles in patients with sorafenib experienced advanced hepatocellular carcinoma (HCC) in phase 2 clinical studies [12, 13]. Another anti-PD-1 monoclonal antibody camrelizumab showed an objective response rate (ORR) of 14.7%, a 6-month overall survival (OS) rate of 74.4%, and a disease control rate (DCR) of 44.2% in a phase 2 study [14]. Based on the study, the China National Medical Products Administration (NMPA) approved camrelizumab as second-line standard of care for advanced HCC. Except for the common irAEs, a special irAE, namely, reactive cutaneous capillary endothelial proliferation (RCCEP), has been observed in patients treated with camrelizumab. This study reported the occurrence and clinicopathological characteristics of RCCEP, as well as its correlation with efficacy of camrelizumab.

## Methods

### Study design and patients

Data of this study was derived from a multicenter, randomized, open-label, two administration methods parallel-group, phase 2 clinical trial assessing camrelizumab in pre-treated advanced HCC patients that was conducted at 13 study sites in China. From November 15, 2016 to November 16, 2017, 217 patients received camrelizumab 3 mg/kg either every 2 weeks (q2w) or every 3 weeks (q3w) and were included in this analysis. The main inclusion criteria included a histological or cytological diagnosis of advanced HCC; progressed on or were intolerant to prior systemic treatment, mainly with sorafenib and/or oxaliplatin-based chemotherapy; not amenable to surgery or local treatment; those with an Eastern Cooperative Oncology Group (ECOG) performance status of 0–1; Child-Pugh scores of 7 or less; at least one measurable lesion as defined by Response Evaluation Criteria in Solid Tumors (RECIST) version 1.1.

### Outcome and assessments

Tumor response was assessed, both by investigators and blinded independent central review (BICR), as complete response (CR), partial response (PR), stable disease (SD), and progressive disease (PD) based on the RECIST version 1.1. ORR that was defined as the proportion of patients with CR or PR, and DCR that was defined the proportion of patients with CR, PR, or SD ≥ 6 weeks. PFS was defined as the time from initial treatment administration to progression or death, whichever occurred first. OS was defined as the time from initial treatment administration to death. AEs were graded according to the National Cancer Institute Common Terminology Criteria for Adverse Events (NCI-CTCAE version 4.03), except RCCEP. The grading criteria for RCCEP was defined as follows: grade 1, nodule(s) with a maximum diameter of ≤ 10 mm, with or without rupture and bleeding; grade 2, nodule(s) with a maximum diameter of > 10 mm, with or without rupture and bleeding; grade 3, generalized nodules throughout the body, which may be complicated by skin infection; grade 4, multiple and generalized nodules, life-threatening condition; and grade 5, death. Occurrence, morphological characteristics, and regression time of RCCEP were closely observed and recorded. RCCEP remission was defined as regressions of all RCCEP lesions.

### Histopathological analysis

The RCCEP lesion tissues were fixed with 10% neutral formaldehyde, dehydrated with an alcohol gradient, infiltrated with paraffin wax, and embedded in paraffin to form paraffin-embedded blocks. Sections (4 μm) were cut for hematoxylin and eosin (H&E) staining, immunohistochemical (IHC), and immunofluorescence staining (Multiplex IF). The sections were baked at 60 °C overnight, and then IHC and Multiplex IF staining were carried out using a Bond RX automatic stainer (Leica Biosystems, Buffalo Grove, IL). The stained sections were scanned using NanoZoomer Digital Pathology System (Nanozoomer 2.0-HT slide scanner (Hamamatsu, Hamamatsu City, Japan). The NDP view imaging software (NDP scan software) was used to analyze the scan results and catch representative images.

### Statistical analyses

Data were analyzed using SAS version 9.4. Median duration of RCCEP was estimated with Kaplan-Meier method, and its 95% confidence interval (CI) was calculated with Brookmeyer-Crowley method. Correlation between RCCEP occurrence and ORR per BICR was analyzed using Fisher’s exact test. Log-rank test was used to compare the PFS or OS distribution between patients who experienced RCCEP and those who did not. Medians were estimated with Kaplan-Meier method, and their 95% CIs were calculated with the Brookmeyer-Crowley method. Cox proportional-hazards model was used to assess the associations of RCCEP occurrence with PFS per BICR and OS and to calculate the hazard ratios (HRs) of PFS and OS for patients with or without RCCEP. Additional Cox model analysis was performed with extrahepatic metastasis (yes vs. no), portal vein invasion (yes vs*.* no), ECOG Performance Status (0 vs. 1), AFP level (≥ 400 ng/ml vs. < 400 ng/ml), and lines of previous treatment (≥ 3 vs. < 3) as covariates. The RCCEP occurrence showed time-dependence, and thus associations of RCCEP occurrence with PFS per BICR and OS were also evaluated using landmark analysis to minimize lead-time bias. For PFS, the cutoff time point considered for landmark analysis was 1.5 months after the first dose. For OS, the cutoff time points considered for landmark analysis were 3.0 months and 5.0 months after the first dose. Landmark survival analyses only included patients who had not experienced PFS or OS events as of the cutoff time points. Log-rank test was used to compare the PFS or OS distribution between patients who experienced RCCEP and those who did not as of the cutoff time point, and Cox proportional-hazards model was also used.

## Results

### RCCEP occurrence, therapeutical approaches, and outcome

With a median duration of follow-up of 12.5 months (range, 0.7–23.5 months), 145 of 217 patients (66.8%) experienced RCCEP, the most common AE related to camrelizumab. All RCCEP were grade 1 (117 patients [53.9%]) or 2 (28 [12.9%]). Among the 217 patients, 106 (48.8%) had the first RCCEP occurrence during the first treatment cycle, whereas only 39 (18.0%) had the first occurrence of during the second cycle or later. The median time to onset of RCCEP was 4.1 weeks (range, 0.1–29.1). There were no reports of visceral bleeding or death caused by RCCEP. No patients interrupted or discontinued treatment due to RCCEP.

Only in rare cases, RCCEP could be complicated by reactive capillary endothelial proliferation in other sites, including oral cavity (three [1.4%]; two in the oral mucosa and one in the gingivae), eyes (two [0.9%]; conjunctiva), and nasal cavity (two [0.9%]; nasal mucosa) (see Additional file 1). The majority of reactive capillary endothelial proliferation in sites other than skin were grade 1 or 2, except one (0.5%), grade 3 reactive capillary endothelial proliferation in the nasal mucosa (see Additional file 1). No patients discontinued camrelizumab due to reactive capillary endothelial proliferation in sites other than skin. Only one patient experienced treatment interruption due to capillary endothelial proliferation in oral mucosa (grade 2).

Totally, 31 of the 145 patients (21.4%) received symptomatic therapy for RCCEP, including laser therapy (18, 12.4%), minor resection (14, 9.7%), hemostatic therapy (nine, 6.2%), local hormone therapy (six, 4.1%), systemic antibiotic treatment (two, 1.4%), and cryotherapy (one, less than 1%).

The median duration of RCCEP was 5.6 months (range, 0.9–22.7). As of the data cutoff date, RCCEP in 86 of the 145 patients (59.3%) achieved remission, with a median time to remission of 4.0 months (range, 0.9–17.1). In 26 of the 86 patients (30.2%), all RCCEP lesions regressed on camrelizumab treatment, while in 60 patients (69.8%), at least one RCCEP lesion regressed or necrosed and fell off after discontinuation of camrelizumab treatment with a median duration from last dose to remission was 1.6 months (range, 0.3–5.3). Among the 145 patients who experienced RCCEP, 32 patients (22.1%) had RCCEP at the date of last dose and did not had a record of RCCEP remission at date cutoff.

### Morphological classification and characteristics of RCCEP

**Fig. 1:**
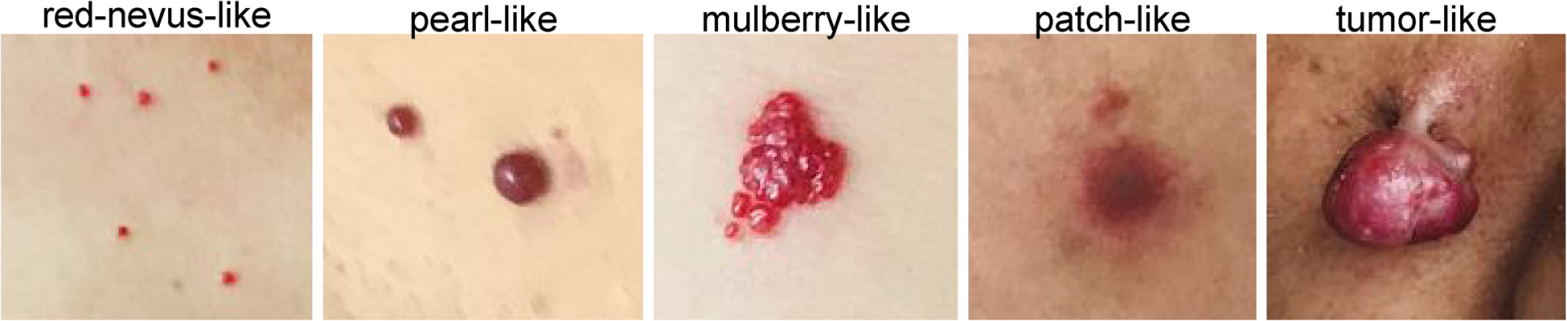
Morphological classification of reactive cutaneous capillary endothelial proliferation

RCCEP occurred on the skin surface, mainly on the skin of the head, face, and trunk. It could be divided into 5 types according to the morphology, namely, “red-nevus-like,” “pearl-like,” “mulberry-like,” “patch-like,” and “tumor-like,” with “red-nevus-like” and “pearl-like” being the most common types (Fig. 1). Various types of RCCEP could be observed in the same patient. Initially, the lesions were usually bright red spots with a diameter of ≤ 2 mm (“red-nevus-like”). A few lesions were “patch- or mulberry-like,” and some “red-nevus-like” lesions gradually developed into “pearl-like” nodules with bright or dark red coloration, which tended to rupture and bleed, while a few of these “pearl-like” nodules developed into “tumor-like” nodules (diameter > 10 mm). Most patients experienced RCCEP 2 to 4 weeks after the first dose. As the dose of medication increased, nodules became larger, increased in number, and gradually expanded in scope. Most skin nodules ceased to increase 3 to 4 months after the first dose. Some nodules gradually shrank, dried, and turned black. Some formed pedicled nodules that fell off spontaneously without leaving obvious scars.

### Pathological features of RCCEP

**Fig. 2:**
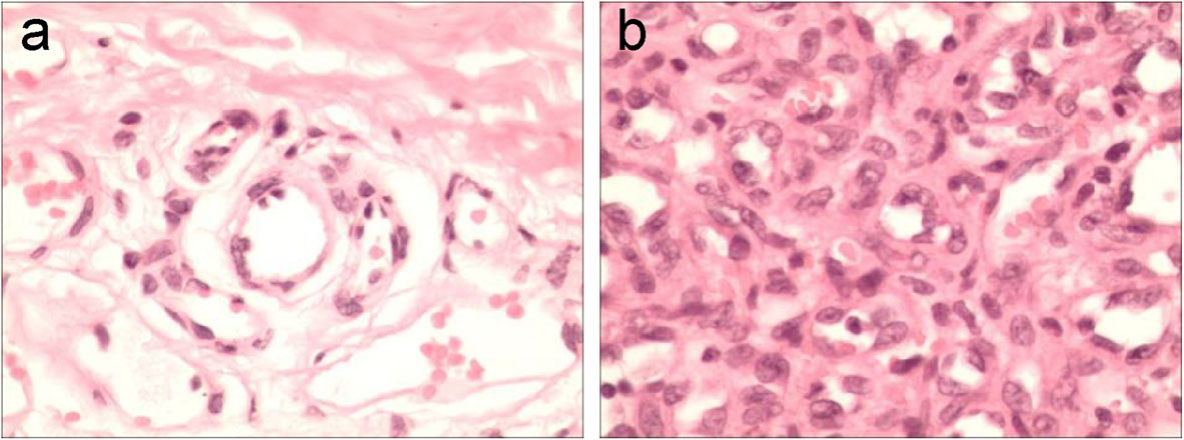
Pathological characteristics of RCCEP. **a** “red-nevus-like” RCCEP pathological tissue. **b** “pearl-like” or “tumor-like” RCCEP pathological tissue. Tissues were analyzed using hematoxylin and eosin (H&E) staining (× 400). RCCEP, reactive cutaneous capillary endothelial proliferation

**Fig. 3:**
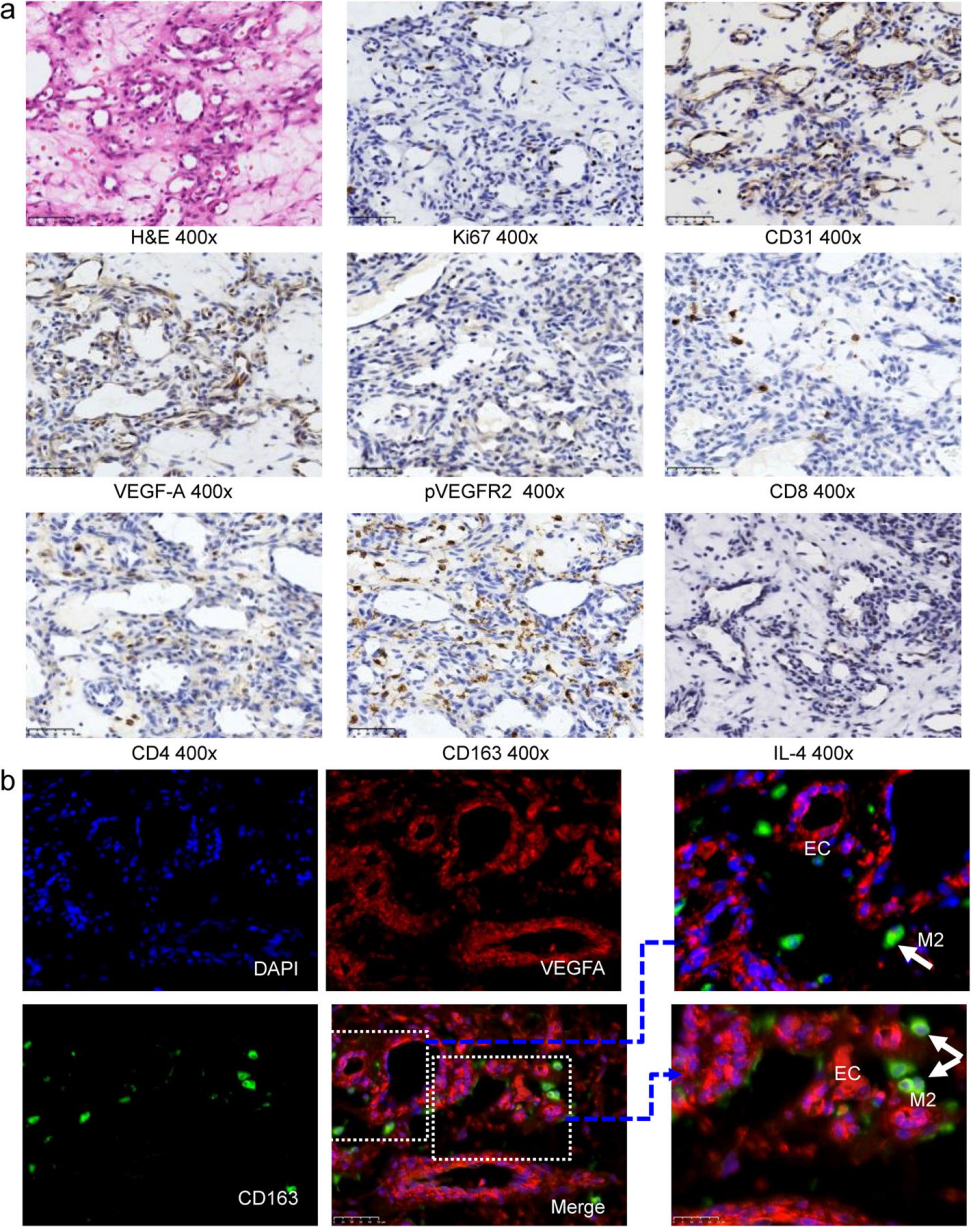
“Pearl-like” RCCEP skin nodule histopathology and molecular marker detection. **a** Immunohistochemistry. **b** Immunofluorescence co-staining (red, VEGF-A; green, CD163; and blue, DAPI. Arrow: M2 macrophages expressing VEGF-A). RCCEP, reactive cutaneous capillary endothelial proliferation

The histopathological features of “red-nevus-like” lesions were as follows: the lesions were located in the reticular layer of dermis; the hyperplastic capillaries were sparsely arranged; and the lining endothelial cells were enlarged without atypia and were all mono-layered (Fig. 2a). The pathological manifestations of “pearl- or tumor-like” lesions were as follows: the lesions were located in the reticular layer of dermis and consisted of hyperplastic capillaries; the capillaries were arranged in lobulated or nodular pattern; fibrous interlobular connective tissue and intralobular or interlobular nutrient vessels with a large lumen were found in some cases; and interstitial fibrosis occurred in some cases (Fig. 2b). IHC staining showed intense staining of endothelial cells (CD31) as well as proliferation and division of endothelial cells (Ki67). High expressions of vascular endothelial growth factor-A (VEGF-A) and VEGFR2-pY1175 in the lesion tissues were detected (Fig. 3a). IF co-staining on the RCCEP biopsy tissue showed that a large number of CD4^+^ T cells, not CD8^+^ T cells, appeared around the capillaries of the lesion tissue. There was a high expression of the Th2 cytokine IL-4. CD163^+^ M2 macrophages were detected in the lesion tissue, and co-location of VEGF-A and CD163^+^ M2 macrophages was identified (Fig. 3b).

### Correlation between RCCEP and the efficacy of camrelizumab

Per BICR, 28 of 145 patients with RCCEP achieved PR. However, among the 72 patients without RCCEP, only 4 achieved PR. No patient achieved CR. Significant association between RCCEP occurrence with higher ORR was observed (19.3% [95% CI, 13.2–26.7] vs. 5.6% [95% CI, 1.5–13.6]; one-sided *p* = 0.0044). The ORR was 28.6% (95% CI, 13.2–48.7) in patients who had grade 2 RCCEP and 17.1% (95% CI, 10.8–25.2) in patients who had grade 1 RCCEP.

**Fig. 4:**
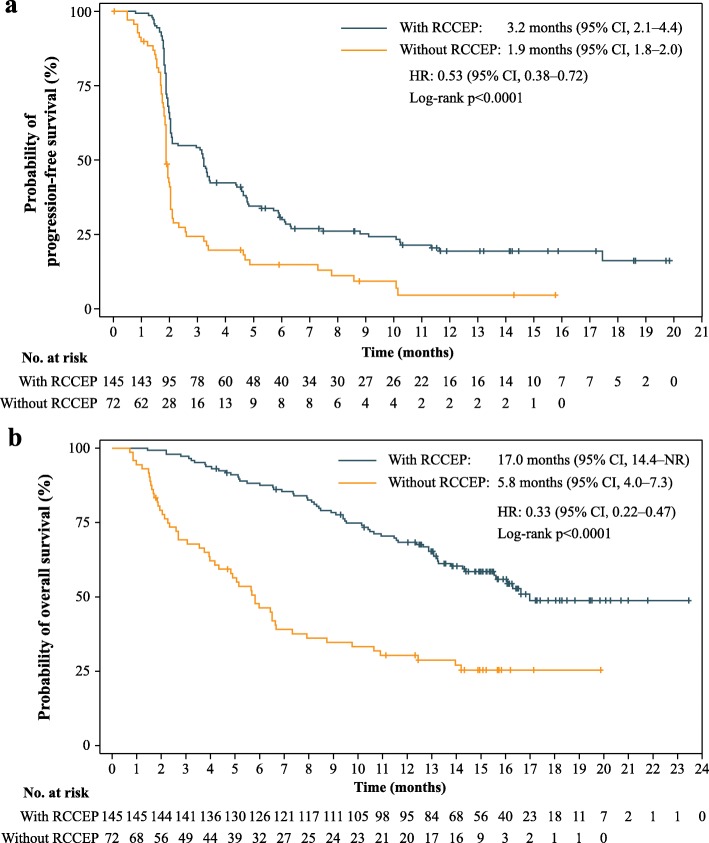
Comparisons in survival between patients with and without RCCEP. **a** Kaplan-Meier curve of PFS. **b** Kaplan-Meier curve of OS. All 217 patients treated with camrelizumab were included in the analysis. RCCEP, reactive cutaneous capillary endothelial proliferation; PFS, progression-free survival; OS, overall survival

Significant associations between RCCEP occurrence with prolonged PFS and OS were observed. The median PFS was 3.2 months (95% CI, 2.1–4.4) in patients with RCCEP and 1.9 months [95% CI, 1.8–2.0] in patients without RCCEP (HR, 0.53 (95% CI, 0.38–0.72) for progression or death; one-sided log-rank test *p* < 0.0001; Fig. 4a). The median OS was 17.0 months (95% CI, 14.4–not reached) in patients with RCCEP and 5.8 months [95% CI, 4.0–7.3] in patients without RCCEP (HR, 0.33 (95% CI, 0.22–0.47) for death; one-sided log-rank test *p* < 0.0001; Fig. 4b). The median OS in patients who had grade 1 RCCEP was 16.1 months (95% CI, 12.9–not reached), while that in patients who had grade 2 RCCEP was not reached (95% CI, 16.2 months–not reached).

Additional Cox model analysis showed that RCCEP occurrence was still significantly correlated with PFS and OS after adjustment for baseline variables (HR for progression or death, 0.53 [95% CI, 0.39–0.73]; HR for death, 0.33 [95% CI, 0.23–0.47).

**Fig. 5:**
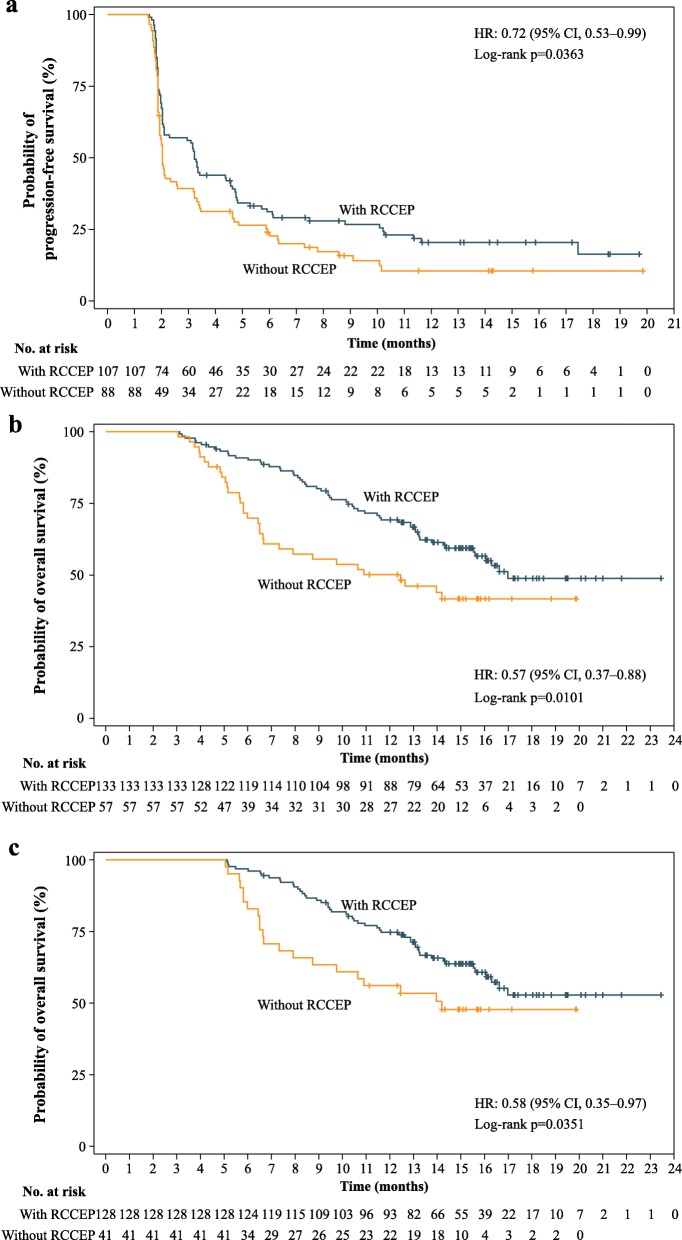
Landmark analysis of survival between patients with and without RCCEP. **a** Landmark survival analysis curve with 1.5 months as the cutoff time point. One hundred and ninety-five patients with PFS ≥ 1.5 months were included in the analysis. The RCCEP group consisted of patients with RCCEP within 1.5 months and the group without RCCEP consisted of patients without RCCEP within 1.5 months. **b** Landmark survival analysis curve with 3.0 months as the cutoff time point. One hundred and ninety patients with OS ≥ 3.0 months were included in the analysis. The RCCEP group consisted of patients with RCCEP within 3.0 months, and the group without RCCEP consisted of patients without RCCEP within 3.0 months. **c** Landmark survival analysis curve with 5.0 months as the cutoff time point. One hundred and sixty-nine patients with OS ≥ 5.0 months were included in the analysis. The RCCEP group consisted of patients with RCCEP within 5.0 months, and the group without RCCEP consisted of patients without RCCEP within 5.0 months. RCCEP, reactive cutaneous capillary endothelial proliferation; PFS, progression-free survival; OS, overall survival

The median duration of camrelizumab treatment in patients with vs. without RCCEP was 5.5 months (range, 0.7–23.5) vs. 1.5 months (range, less than 0.1–16.2). Landmark survival analyses were conducted to take account of the time-dependence nature of RCCEP. The results further supported the significant associations between the RCCEP occurrence with PFS and OS. The landmark analysis of PFS at 1.5 months showed that the HR for progression or death of patients with RCCEP vs*.* patients without RCCEP at 1.5 months was 0.72 (95% CI, 0.53–0.99; one-sided log-rank test *p* = 0.0363; Fig. 5a). Landmark survival analysis of OS at 3 and 5 months showed that the HRs for death of patients with RCCEP vs. patients without RCCEP at 3 and 5 months were 0.57 (95% CI, 0.37–0.88; one-sided log-rank test *p* = 0.0101; Fig. 5b) and 0.58 (95% CI, 0.35–0.97; one-sided log-rank test *p* = 0.0351; Fig. 5c), respectively.

## Discussion

In recent years, a number of clinical studies have been conducted on the treatment of advanced HCC with anti-PD-1/PD-L1 monoclonal antibodies. Nivolumab and pembrolizumab have been conditionally approved by the U.S. Food and Drug Administration as second-line treatments for advanced HCC. However, the KEYNOTE-240 phase 3 study found no statistical difference in OS and PFS between pembrolizumab and placebo combined with the best support treatment [15]. The CheckMate-459 study was a phase 3 study of nivolumab vs. sorafenib as a first-line treatment for advanced HCC, and there was no statistical difference between the two therapies in the primary endpoint (i.e., OS) [16]. It is encouraging that the results of the IMbrave150 phase 3 study comparing atezolizumab (an anti-PD-L1 antibody) plus bevacizumab vs. sorafenib as first-line treatment are positive (median OS, not reached with the combination therapy vs. 13.2 months with sorafenib, *p* = 0.0006; median PFS, 6.8 vs. 4.3 months, *p* < 0.0001) [17]. Combination of pembrolizumab and lenvatinib as well as nivolumab and ipilimumab in advanced HCC also has achieved encouraging results [18, 19].

ICIs can cause irAEs in multiple organs, with skin reactions being the most common. The skin irAEs approximately occur in 34% of patients treated with nivolumab or pembrolizumab [20] and usually do not require treatment discontinuation or dose reduction. Serious skin irAEs are rare. Besides common skin irAEs such as rash and pruritus, RCCEP is an irAE related to camrelizumab with the highest incidence. In the phase 1 clinical studies of camrelizumab monotherapy for the treatment of esophageal and nasopharyngeal carcinomas [21, 22], incidence of RCCEP were 76.7% (23/30) and 88% (82/93), respectively, but all events were grade 1 or 2 and did not lead to treatment discontinuation.

In this study, incidence of RCCEP was 66.8% (145/217), and the events mainly occurred on the skin of the head, face, and trunk. RCCEP is different from common skin rash, which has patchy distributions. Most of the RCCEP lesions were isolated and scattered on the skin. It is difficult to adopt the traditional grading criteria based on the covering area of body surface. Therefore, the grading standard for RCCEP was proposed based on the results of clinical studies and observation in clinical practices. This grading criterion has been adopted by the China NMPA and included in the label for camrelizumab. In this study, all RCCEP were grades 1 and 2, and no grades 3–5 RCCEP were observed.

Cutaneous hemangioma is a benign vasculopathy with various classifications. It usually occurs in infants, at birth or within 1 month after. It mostly invades the skin of the head and neck, but can also occur in the mucosa, liver, lower limbs, and muscles. It is sensitive to glucocorticoid treatment, and a few cases will spontaneously resolve after several months or years. RCCEP is different from common hemangioma in terms of pathogenesis, morphological manifestations, pathological features, treatment outcomes, and ways of remission. Therefore, RCCEP cannot be defined by the term “hemangioma.” Pathological analysis of early-stage RCCEP showed an increased size of capillary endothelial cells and capillary hyperplasia in dermis; when it developed to “pearl-like” or “tumor-like” RCCEP, the capillaries obviously increased and showed a lobulated or nodular arrangement. Based on the IHC analysis, strong staining of CD31 indicated a large number of endothelial cells in the lesion tissue, and high expression of Ki-67 indicated rapid proliferation of endothelial cells. High expressions of VEGF-A and VEGFR2-pY1175 demonstrated activation of angiogenesis via VEGFR2 signal pathway. Combined with the results of IF, camrelizumab might activate CD4^+^ T cells, thus increasing Th2 cytokine IL-4, stimulating the differentiation of CD163^+^ M2 macrophages, and then promoting vascular proliferation by releasing VEGF-A.

Anti-VEGFR2 antibodies such as ramucirumab, tanibirumab, and CDP791 could cause grades 1 and 2 cutaneous capillary hyperplasia in clinical studies and lesions regressed after treatment were stopped. The VEGF-A concentration increased, and VEGFR-2 was widely expressed in the specimens of lesion tissues [23–25]. Hwang et al. reported that 49% (40/82) of patients with metastatic melanoma treated with nivolumab or pembrolizumab experienced skin AEs, among whom 2 cases experienced cutaneous capillary hyperplasia (2.4%) [26]. Therefore, RCCEP may not be unique to camrelizumab, but with a high incidence and different manifestations. It is speculated that the reactivation of immune responses may destroy the dynamic balance between pro- and anti-angiogenic factors, and thus cause proliferation of skin capillary endothelial cells.

In a phase 1 study in advanced HCC, gastric cancer, and gastroesophageal junction cancer, the incidence of RCCEP in patients treated with camrelizumab combined with apatinib was only 12.1% (4/33), and all events were grades 1 and 2 [27]. Apatinib is a highly selective VEGFR2 inhibitor. It is presumed that it inhibited the development of RCCEP by blocking signal transduction after binding to VEGF. In a phase 1 study in nasopharyngeal carcinoma, the incidence of RCCEP with camrelizumab monotherapy was 88% (82/93), whereas the incidence with camrelizumab plus gemcitabine/cisplatin decreased to 22% (5/23) [22]. Chemotherapy may inhibit the RCCEP occurrence by affecting angiogenesis.

Shi et al. reported that among the 20 ICI-treated patients who developed skin irAEs, four patients are complicated by oral mucosa lesions involving the tongue, buccal mucosa, lips, and/or gingivae, one showed papules, and three had erosions resembling oral lichen planus [28]. In this study, very few patients with RCCEP had lesions in the oral mucosa, gingivae, nasal mucosa, and palpebral conjunctiva. However, no occurrence in the tracheal or digestive (esophageal and gastrointestinal) mucosa was observed. Therefore, there is no risk of hemoptysis or gastrointestinal hemorrhage.

Skin irAEs could predict the efficacy of anti-PD-1 monoclonal antibodies in some solid tumors [29–31]. In this study, RCCEP occurrence was significantly correlated with response to camrelizumab. ORR was 19.3% vs. 5.6% in patients with vs. without RCCEP. In addition, significant associations were observed between RCCEP occurrence with prolonged PFS and OS, suggesting that patients with RCCEP had better prognoses. Therefore, RCCEP may become a clinical biomarker for predicting efficacy of camrelizumab.

Most RCCEP did not require special treatment. RCCEP was insensitive to glucocorticoids, and most lesions spontaneously resolved within 1.6 months after discontinuation of camrelizumab. If the nodule is large, and rupture and bleeding occur, Yunnan Baiyao powder can be given for local hemostasis, and mupirocin ointment can be given, if necessary, to prevent infection. Minor resection or laser treatment can be adopted for a few patients. If skin infection occurs, local or systemic antibiotic treatment should be conducted. When grade of RCCEP decreases to grade 1, camrelizumab administration can be resumed as appropriate.

The major limitation of this study is that mechanism of RCCEP occurrence is still unclear, but may be attributed to immune stress responses of the cutaneous capillary endothelial cells.

## Conclusion

RCCEP was a common irAE of camrelizumab, which mainly occurred on the skin of the head, face, and trunk. It had unique morphological features and often spontaneously regressed or necrosed and fell off after treatment discontinuation. RCCEP occurrence was closely related to the objective response and PFS and OS benefits of camrelizumab treatment, which requires further investigations.

## Supplementary information

**Additional file 1.** Occurrence site and grade of reactive capillary endothelial proliferation.

## Data Availability

All data generated or analyzed during this study are included in this published article and its supplementary information files.

## Abbreviations

RCCEP: Reactive cutaneous capillary endothelial proliferation
ICI: Immune checkpoint inhibitor
PD-1: Programmed cell death protein-1
HCC: Hepatocellular carcinoma
FDA: Food and Drug Administration
NMPA: National Medical Products Administration
ECOG: Eastern Cooperative Oncology Group
AE: Adverse event
irAE: Immune-related adverse event
BICR: Blinded independent central review
CR: Complete response
PR: Partial response
SD: Stable disease
PD: Progressive disease
ORR: Objective response rate
DCR: Disease control response
PFS: Progression-free survival
OS: Overall survival
CI: Confidence interval
IHC: Immunohistochemistry
IF: Immunofluorescence
AFP: Alpha-fetoprotein
