# Supplementary material for: Reactive cutaneous capillary endothelial proliferation in advanced hepatocellular carcinoma patients treated with camrelizumab: data derived from a multicenter phase 2 trial
Source: J Hematol Oncol. 2020 May 11;13:47. doi: 10.1186/s13045-020-00886-2 (PMC7216554; doi:10.1186/s13045-020-00886-2)
Supplement: Supplementary file 1 — Additional file 1. Occurrence site and grade of reactive capillary endothelial proliferation. [file 13045_2020_886_MOESM1_ESM.docx]

## Additional file 1 Occurrence site and grade of reactive capillary endothelial proliferation

|  | **All treated patients**  **(N=217)** | **Dose frequency** | |
| --- | --- | --- | --- |
|  |  | **Every 2 weeks (N=109)** | **Every 3 weeks (N=108)** |
| Skin (RCCEP) | 145 (66.8) | 69 (63.3) | 76 (70.4) |
| Grade 1 | 117 (53.9) | 54 (49.5) | 63 (58.3) |
| Grade 2 | 28 (12.9) | 15 (13.8) | 13 (12.0) |
| Oral cavity | 3 (1.4) | 2 (1.8) | 1 (0.9) |
| Grade 2 | 3 (1.4) | 2 (1.8) | 1 (0.9) |
| Eyes | 2 (0.9) | 0 | 2 (1.9) |
| Grade 1 | 1 (0.5) | 0 | 1 (0.9) |
| Grade 2 | 1 (0.5) | 0 | 1 (0.9) |
| Nasal cavity | 2 (0.9) | 1 (0.9) | 1 (0.9) |
| Grade 1 | 1 (0.5) | 1 (0.9) | 0 |
| Grade 3 | 1 (0.5) | 0 | 1 (0.9) |

Data are shown in n (%).

RCCEP, reactive cutaneous capillary endothelial proliferation.
